# Acute Heart Failure Exacerbation with Cardiogenic Shock and Elevated Systemic Vascular Resistance Treated with a Combination of Nicardipine and Dobutamine Therapy

**DOI:** 10.1155/2017/7329213

**Published:** 2017-08-06

**Authors:** Lydia E. Issac, Setri Fugar, Naser Yamani, Burhan Mohamedali

**Affiliations:** ^1^Department of Internal Medicine, John H. Stroger Hospital of Cook County, 1969 W Ogden Ave., Chicago, IL 60612, USA; ^2^Department of Internal Medicine, Rush Medical College, Rush University Medical Center, 1653 W. Congress Pkwy, Chicago, IL 60612, USA; ^3^Department of Internal Medicine, Advanced Heart Failure/Transplant Cardiology, Rush University Medical Center, 1653 W. Congress Pkwy, Chicago, IL 60612, USA

## Abstract

Acute heart failure is a common reason for hospital admission and is usually caused by decreased cardiac output either as a result of an intrinsic cardiac issue or as a result of severe hypertension with elevated afterload. We present a patient with a history of HFrEF who presented with acute heart failure, found to have hypotension requiring Dobutamine support and an elevated systemic vascular resistance requiring Nicardipine drip, with subsequent recovery of cardiac function.

## 1. Introduction

Heart failure is a clinical syndrome characterized by a decrease in cardiac output with or without an increase in intracardiac pressure, which can be caused by structural or functional pathologies. One of the determinants of cardiac output (CO) is afterload, which is the force that the heart must overcome to pump blood to the body [1]. Systemic vascular resistance (SVR) is a measure of resistance of systemic vascular bed to blood flow and can be used to clinically monitor left ventricular afterload [2]. An elevated SVR can result in the inability to increase the stroke volume to match the body's demand. In cases wherein the afterload is elevated and the heart cannot generate enough pressure to overcome it, heart failure decompensation can occur. We present a patient with longstanding hypertension who presented with heart failure exacerbation, with hypotension and elevated SVR.

## 2. Case Presentation

A 53-year-old lady with history of HTN, initially characterized by HFpEF which progressed to HFrEF, presented with heart failure exacerbation secondary to medication noncompliance. Vital signs on admission included temperature 98.6F, BP 127/83, HR 92, RR 18, and O2 sat 93% on 2 L nasal cannula. Echocardiogram showed a LVEF of 25–30% with Grade 2 diastolic dysfunction. Intravenous diuresis was initiated with development of hypotension. Dobutamine was started but was unable to be weaned off. Left heart catheterization revealed no evidence of CAD. Right heart catheterization results are shown in Table 1. Nicardipine drip was started as SVR was high. Dobutamine was uptitrated to maintain MAPs above 70 mmHg. Clinical response to the drip is shown under Days 2-3. As her MAPs were stable, she was weaned off Dobutamine by Day 3. Subsequently, Nicardipine drip was weaned off. Her fluid status continued to improve with diuresis. She was discharged and, on follow-up in clinic, is doing well.

## 3. Discussion

Heart failure has a prevalence of over 5.8 million people in the United States and greater than 23 million people around the world [3]. Patients with acute heart failure usually either have uncontrolled HTN with acute pulmonary congestion requiring vasodilator therapy or have hypotension, usually due to an intrinsic cardiac problem requiring inotropic support and/or mechanical assistance.

This patient's profile is a unique blend between a patient with hypertensive emergency presenting with heart failure exacerbation requiring vasodilators such as Nicardipine and someone in a low cardiac output state requiring inotropic support. The likely pathophysiology of this patient's presentation is that she had a “hypertensive emergency,” because of her baseline reduced EF, resulting in the inability to compensate for the elevated SVR, which, in turn, resulted in hypotension. This is in line with the formula for Mean Arterial Pressure: MAP = CO *∗* SVR.

Vasodilators help with heart failure as they decrease left ventricular filling pressures. This can increase cardiac output as the stroke volume will increase due to decrease in SVR and then result in decreased myocardial oxygen demand [4]. Calcium channel antagonists can reduce SVR due to their vasodilatory effect in the short term. However, they are well known for their negative inotropic effects.

Aroney et al. studied the inotropic effect of Nicardipine in acute heart failure. The study included 15 patients with heart failure with reduced ejection fraction (0.15 ± 0.02), NYHA II to IV. These patients received equihypotensive doses of IV Nitroprusside and IV Nicardipine. The patient's baseline values were obtained followed by infusion of Nitroprusside, returning to a second baseline, followed by Nicardipine infusion. LV pressure volume loops showed a rightward shift with Nicardipine, suggestive of negative inotropic effect. Overall this study suggested an increase in Cardiac Index, decrease in SVR, and increase in LV ejection fraction with Nicardipine [5]. A study by Hirota et al. also showed increased cardiac output with Nicardipine infusion in patients with HFrEF exacerbation [6].

Management of patients with such heart failure requires the use of vasodilators which help increase CO by decreasing SVR, with the aid of inotropes to maintain the BP as the SVR decreases. The above combination helped our patient to avoid chronic inotropic support and further advanced heart failure care including mechanical assist devices or heart transplant. To the best of our knowledge, this is the first case in which a patient with acute heart failure exacerbation with cardiogenic shock benefited from the combination of Dobutamine for inotropic support and Nicardipine for improvement in SVR.

## 4. Conclusion

In patients with a known history of hypertensive heart disease presenting with acute heart failure and cardiogenic shock, a low cardiac output due to increased systemic vascular resistance may exist. Measurement of the SVR may lead to the use of vasodilator therapy, in combination with inotropes, which may lead to resolution of the heart failure.

## Figures and Tables

**Table 1:** Right heart catheterization results and corresponding days.

	Day 1	Day 2	Day 3
Central Venous Pressure (CVP)	15	6	8
Cardiac output (CO)	3.5	5.2	5.3
Cardiac Index (CI)	1.9	2.8	2.8
Pulmonary Vascular Resistance (PVR)	228	175	136
Systemic Vascular Resistance (SVR)	1965	1169	87.5
Stroke Volume (SV)	33.3	65	54.1
